# Necroptosis in SJS/TEN: *RIPK1* and *RIPK3* Expression and Implications for Disease Pathogenesis

**DOI:** 10.3390/cimb48050540

**Published:** 2026-05-21

**Authors:** Chandana Sooranahalli, Charles Bouchard, Omer Iqbal

**Affiliations:** 1 Department of Ophthalmology, Stritch School of Medicine, Loyola University Chicago, Maywood, IL 60153, USA; csooranahalli@luc.edu; 2 Department of Ophthalmology, Loyola University Medical Center, Maywood, IL 60153, USA; cboucha@lumc.edu; 3 Departments of Ophthalmology & Pathology, Stritch School of Medicine, Loyola University Chicago, Maywood, IL 60153, USA

**Keywords:** Stevens–Johnson syndrome (SJS), toxic epidermal necrolysis (TEN), SJS/TEN pathogenesis, necroptosis, severe cutaneous adverse reactions (SCARs), RIPK1, RIPK3, cytokines in SJS/TEN, immune response in skin disorders, inflammatory pathways in SJS/TEN, cell death pathways

## Abstract

Necroptosis has been implicated in the pathogenesis of Stevens–Johnson syndrome and toxic epidermal necrolysis (SJS/TEN), with prior studies demonstrating tissue-level involvement of receptor-interacting protein kinases RIPK1 and RIPK3. However, their systemic expression in the circulatory compartment remains incompletely characterized. The objective of this study is to evaluate circulating levels of RIPK1 and RIPK3 in patients with SJS/TEN and explore their potential association with diseases. Serum samples from patients with SJS/TEN and control groups were analyzed for RIPK1 and RIPK3 levels using ELISA. Group differences were assessed using non-parametric statistical methods. Circulating levels of RIPK1 and RIPK3 were elevated in patients with SJS/TEN compared with controls. These findings were consistent across analyses; however, variability within groups and overlap between cohorts were observed. These results suggest an association between increased circulating RIPK1 and RIPK3 levels and SJS/TEN. Given the limited sample size, heterogeneous control populations, and lack of functional or phosphorylation-specific assays, these findings should be considered exploratory. Further studies incorporating larger cohorts and mechanistic validation are needed to clarify the role of necroptosis-related pathways in the systemic manifestations of SJS/TEN.

## 1. Introduction

Stevens–Johnson syndrome (SJS) and toxic epidermal necrolysis (TEN) represent a continuum of severe cutaneous adverse reactions characterized by widespread epidermal necrosis, mucocutaneous detachment, and significant systemic inflammation [1]. SJS/TEN is rare, with an estimated incidence of 5–6 cases per million annually, but carries the risk of substantial morbidity and mortality, ranging from 1–5% in SJS to as high as 20–30% in TEN [2]. Survivors frequently experience chronic complications involving the ocular surface, respiratory tract, urogenital epithelium, and gastrointestinal mucosa, with ocular sequelae such as chronic dry eye, conjunctival scarring, and vision loss among the most disabling outcomes [3]. Given the rapid progression and high risk of multisystem failure, it is critical that we prioritize early identification and mechanistic understanding of SJS/TEN.

SJS/TEN commonly arises as the result of an idiosyncratic, delayed-type hypersensitivity reaction to medications. High-risk drugs include anticonvulsants, sulfonamide antibiotics, allopurinol, NSAIDs of oxicam origin, and certain antiretrovirals [4,5,6,7]. Specific HLA alleles, such as *HLA-B1502* with carbamazepine and *HLA-B5801* with allopurinol, have strong associations with SJS/TEN, pointing to a degree of genetic susceptibility. Identification of these genetic susceptibilities has enabled population-specific risk stratification and even pre-prescription screening in some regions [4,5,6,7]. However, despite these advances in risk identification, the mechanisms linking drug exposure and host immune activation to widespread epithelial injury largely remain a mystery.

Broadly, SJS/TEN is initiated by the activation of drug-specific cytotoxic CD8+ T cells and natural killer (NK) cells, which infiltrate the skin and mucosal surfaces and induce keratinocyte death through perforin, granzyme B, and granulysin release [8,9]. In parallel, a surge of pro-inflammatory cytokines, including TNF-α, IL-6, IL-15, and IL-33, transmit signals for tissue damage and systemic inflammatory responses [10,11,12,13]. Prior studies from our laboratory and others have demonstrated the role of innate immune pathways, acknowledging that cytokines such as IL-1β, IL-33, and TGF-β1 likely influence keratinocyte susceptibility to death signals and the overall microenvironment accompanying inflammation [14,15,16,17]. These observations support the growing consensus that SJS/TEN is driven by a complex mix of adaptive and innate immune mechanisms.

**Figure 1:**
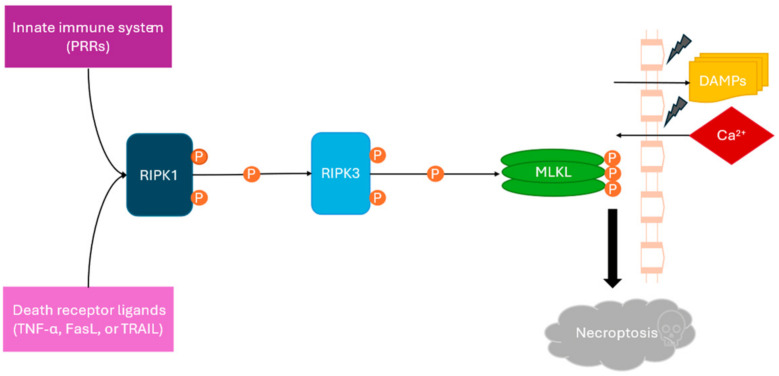
Proposed role of necroptosis in SJS/TEN pathogenesis. Death receptor and innate immune signaling promote RIPK1/RIPK3/MLKL-mediated necrosome formation, resulting in keratinocyte death, epidermal injury, DAMP release, and inflammatory amplification through mitochondrial ROS and inflammasome activation.

Historically, keratinocyte death in SJS/TEN was attributed largely to apoptosis mediated by Fas–FasL signaling, cytotoxic granule pathways, and caspase activation [18]. However, increasing evidence indicates that apoptosis alone cannot account for the extensive and rapid epidermal detachment characteristic of TEN. Instead, recent work has implicated necroptosis, a regulated form of necrotic cell death, as a key mediator of epithelial destruction [19,20,21,22]. Necroptosis is driven by receptor-interacting serine/threonine-specific protein kinases 1 and 3 (RIPK1 and RIPK3), which assemble into a multiprotein necrosome and promote phosphorylation of mixed lineage kinase domain-like protein (MLKL). Activated MLKL disrupts cell membranes, releasing damage-associated molecular patterns (DAMPs), an influx of extracellular calcium ions, mitochondrial reactive oxygen species (ROS), and ATP that amplify inflammation through NLRP3 inflammasome activation and cytokine maturation ([23,24,25,26,27]; Figure 1).

Recent studies have demonstrated upregulation of RIPK3 and MLKL phosphorylation in epidermal samples from patients with SJS/TEN, suggesting that necroptosis contributes significantly to keratinocyte death and inflammatory amplification [25]. Our previous work has similarly shown elevated levels of IL-1β, IL-6, RIPK1, and other cytokines in tissue biopsy samples of SJS/TEN patients, further implicating innate immune activation and inflammasome signaling in disease pathogenesis [28,29,30,31,32,33,34,35]. Together, these findings support a model in which necroptosis acts alongside apoptosis to drive widespread epithelial injury, systemic inflammation, and multi-organ involvement.

Despite this emerging evidence, relatively little is known about systemic markers of necroptotic activation in SJS/TEN. Most studies have focused on tissue biopsies, leaving unanswered questions about whether necroptosis can be detected in the circulation and whether circulating RIPK1 and RIPK3 reflect disease severity or systemic inflammatory burden.

The present study addresses this knowledge gap by evaluating plasma levels of RIPK1 and RIPK3 in patients with biopsy-confirmed SJS/TEN, comparing them with both normal and pathologic pooled plasma controls. By integrating these findings with prior evidence from tissue studies and cytokine profiling, we aim to clarify the role of necroptosis in systemic disease progression. The goal is not to establish diagnostic or prognostic utility but to determine whether necroptosis-associated kinases implicated in tissue injury are detectable systemically.

## 2. Materials and Methods

The de-identified and discarded plasma samples from patients with SJS/TEN were obtained from the Loyola University core labs. Under the current Loyola IRB protocol, deidentified platelet-poor plasma samples of 22 patients from the Loyola core pathology lab were obtained.

Study samples were stratified into three predefined groups: Stevens–Johnson syndrome/toxic epidermal necrolysis (SJS/TEN), positive controls (inflammatory disease controls with confirmed systemic immune activation), and negative controls (healthy individuals without known acute inflammatory or autoimmune conditions). Control-group assignment was based on clinical diagnosis and chart reviews prior to sample analysis to minimize classification bias.

All plasma samples were stored at −70 °C and contained either EDTA or citrate as an anticoagulant. Normal human plasma (*n* = 3) and pathologic pooled plasma (*n* = 3) were used as negative controls. Clinical metadata including the SCORTEN score, the time from symptom onset to blood draw, the culprit medication, and treatment exposure were not available due to de-identification, which limits clinical correlation.

The pathological pooled plasma used as a positive control consisted of commercially available or laboratory-aggregated plasma derived from individuals with underlying inflammatory or disease states. While these samples were selected to provide a comparator reflecting non-healthy physiological conditions, detailed clinical characterization of these pooled controls (including specific diagnoses, treatments, and inflammatory profiles) was not available. As such, these controls represent a heterogeneous group and may not fully recapitulate disease processes comparable to SJS/TEN.

ELISA analysis of plasma samples was performed using a Human RIPK1 Colorimetric kit (Novus Biologicals, Centennial, CO, USA) and a Human RIPK3 Colorimetric kit (Novus Biologicals, Centennial, CO, USA). All reagent preparations and ELISA experimentation were performed according to the manufacturer’s instructions. These human RIPK1 and RIPK3 ELISA kits include a set of calibration standards. The calibration standards were assayed at the same time as the samples and allowed the operator to produce a standard curve of optical density based on human RIPK1 and RIPK3 concentrations. The concentration of the samples is then determined by comparing the O.D. of the samples to the standard curve.

Both RIPK1 and RIPK3 plates were analyzed on a SpectraMax ABS microplate reader (Molecular Devices, Sunnyvale, CA, USA) set at 450 nm. Optical density (OD) values were analyzed using GainData^®^ (arigo ELISA Calculatorarigo Biolaboratories Corp., Hsinchu City, Taiwan)). A four-parameter logistic (4PL) model was applied to fit the standard curve according to the equation

y = D + (A − D)/[1 + (x/C)^B^]

where y represents the mean OD, x is the analyte concentration, A and D represent the minimum and maximum asymptotes, C is the inflection point (EC_50_), and B is the slope factor. Standard concentrations (S1–S8) ranged from 0 to 10 ng/mL and were measured in triplicate. Curve fitting was performed using log_10_-transformed concentrations with automatic weighting in GainData^®^. Assay performance was evaluated using the linearity and goodness-of-fit tests of the standard curve (R^2^ reported for each analyte). The lower and upper limits of quantification (LLOQ and ULOQ) were defined based on the lowest and highest standard concentrations with acceptable accuracy and precision. Intra-assay variability was assessed across triplicates, and values with high variability were reviewed for technical error prior to inclusion.

The concentrations of unknown samples (U1–U22) and controls (C1–C2) were interpolated from the standard curve using their mean OD values. Back-calculated concentrations for standards met the acceptance criteria of ≤20% coefficient of variation (CV) and ≤20% relative error (RE), confirming plate validity. The lower limit of quantification (LLOQ) corresponded to the lowest standard (S8 = 0 ng/mL) meeting these criteria, and the upper limit (ULOQ) corresponded to S1 = 10 ng/mL.

Statistical analyses were performed using GraphPad Prism (version 10.0; GraphPad Software, San Diego, CA, USA). Data were log-transformed prior to analysis.

Group comparisons were performed using non-parametric tests due to the non-normal distribution of biomarker concentrations. Overall differences across groups were assessed using Kruskal–Wallis tests. Effect sizes were estimated using epsilon-squared (ε^2^) to quantify the proportion of rank variance explained by group membership.

Post hoc pairwise comparisons were conducted using Mann–Whitney *U* tests with significance thresholds adjusted for multiple comparisons. Effect sizes for pairwise comparisons were reported using rank-biserial correlation, and median differences were estimated using the Hodges–Lehmann estimator with 95% confidence intervals. Confidence intervals were calculated using bootstrap resampling where appropriate.

All statistical tests were two-sided, with *p* < 0.05 considered statistically significant.

## 3. Results

A total of 44 deidentified plasma samples were analyzed, including 22 samples from patients with biopsy-confirmed Stevens–Johnson Syndrome, SJS/TEN overlap, or TEN, in addition to pathologic pooled plasma and normal human plasma controls. All samples were processed using standardized ELISA protocols with 4-parameter logistic (4PL) curve fitting.

**Figure 2:**
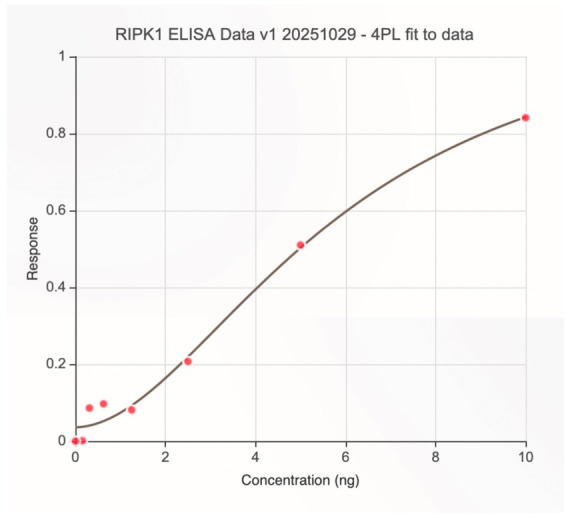
RIPK1 ELISA standard curve fitted with a 4-parameter logistic model (R^2^ = 0.998), showing triplicate mean standards (0–10 ng/mL), with LLOQ and ULOQ indicated by dashed lines; error bars are within the symbol size.

**Figure 3:**
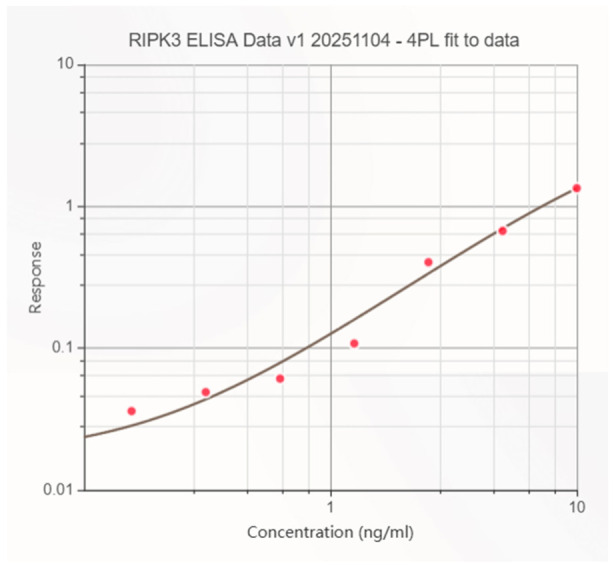
RIPK3 ELISA standard curve fitted with a 4-parameter logistic model (*R^2^* = 0.993), showing mean ± SD of triplicate standards (0–10 ng/mL), with LLOQ and ULOQ indicated; error bars are within the symbol size.

Both RIPK1 and RIPK3 ELISA standard curves demonstrated a high degree of analytical performance, each exhibiting a characteristic sigmoidal 4PL response with high goodness of fit (R^2^ = 0.998 for RIPK1; R^2^ = 0.993 for RIPK3) [Figure 2 and Figure 3]. All calibration standards fell within ±15% of the expected concentrations, meeting standard bioanalytical acceptance criteria [16,17]. No evidence of plate saturation or the hook effect was observed at the highest standard concentrations, confirming suitability of the assay range (0–10 ng/mL).

**Figure 4:**
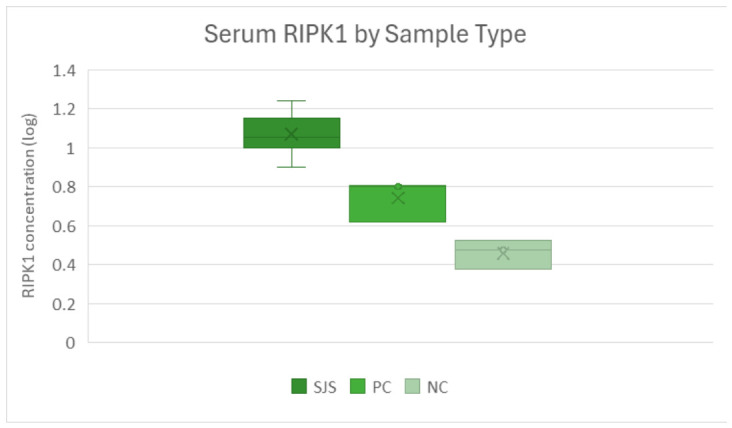
Box-and-whisker plot of serum RIPK1 by sample type. Mean log-transformed RIPK1 concentrations were 1.11 ng (SJS), 0.742 ng (positive control, PC), and 0.536 ng (negative control, NC). RIPK3 trends mirrored those of RIPK1. SJS/TEN samples demonstrated the highest mean log-transformed concentration (0.878 ± 0.247), compared with positive controls (0.675 ± 0.180) and negative controls (0.610 ± 0.190).

Log-transformed RIPK1 concentrations differed across groups, with statistically significant differences observed in overall comparison testing. SJS/TEN samples exhibited the highest mean concentration (1.11 ± 0.143), followed by positive controls (0.742 ± 0.104) and negative controls (0.536 ± 0.212) [Figure 4].

A Kruskal–Wallis test demonstrated a significant overall group effect (H = 9.27, *p* = 0.0097), corresponding to a small-to-moderate effect size (ε^2^ = 7.27/(n − 3)). This indicates meaningful group-level variation in circulating RIPK1 concentrations.

Post hoc pairwise Mann–Whitney U testing showed that SJS/TEN samples had significantly higher RIPK1 concentrations than negative controls (*p* = 0.012), with a consistent direction of effect. The comparison with positive controls did not reach statistical significance (*p* = 0.064), although the directionality of the effect was preserved, suggesting limited power rather than an absence of effect.

Group differences were statistically significant (H = 8.93, *p* = 0.0115), corresponding to a small-to-moderate effect size (ε^2^ = 6.93/(*n* − 3)).

**Figure 5:**
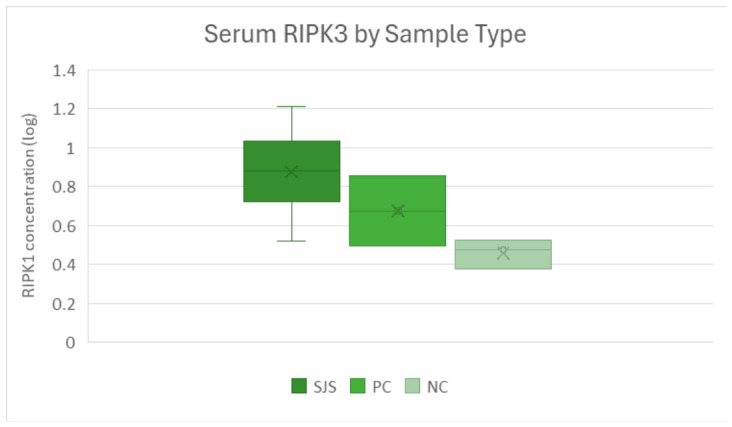
Box-and-whisker plot of serum RIPK3 by group, showing highest levels in SJS (0.878 ng), followed by positive controls (0.675 ng) and negative controls (0.610 ng) on log-transformed values.

Pairwise testing demonstrated significantly higher RIPK3 levels in SJS/TEN compared with negative controls (*p* = 0.011) [Figure 5]. The comparison between SJS/TEN and positive controls approached significance (*p* = 0.058), with a consistent directional trend.

Although SJS/TEN samples showed a clear upward shift in both RIPK1 and RIPK3 concentrations, inter-sample variability was observed across groups. Intra-assay coefficients of variation were <15%, supporting analytical reproducibility and suggesting that observed variability reflects biological rather than technical heterogeneity.

In summary, plasma levels of RIPK1 and RIPK3 differed significantly across study groups, with consistent effect directionality and small-to-moderate effect sizes supporting increased circulating levels in SJS/TEN compared with controls. However, comparisons with pathological plasma controls showed weaker effects and did not consistently reach statistical significance, highlighting biological heterogeneity and the limited sample size.

## 4. Discussion

This exploratory study demonstrates that necroptosis-associated kinases RIPK1 and RIPK3 are detectable and elevated in the plasma of patients with biopsy-confirmed SJS/TEN compared with controls. These findings extend prior tissue-based observations and suggest that severe mucocutaneous injury in SJS/TEN is associated with increased circulating levels of necroptosis-associated proteins [19,20,21,22,23].

Importantly, elevated levels of circulating RIPK1 and RIPK3 cannot be interpreted as evidence of active necroptosis. The ELISA assays used measured total protein rather than phosphorylated (active) forms and therefore do not provide direct evidence of pathway activation. Elevated circulating levels may reflect generalized inflammation, immune-cell activation, and/or passive release from injured keratinocytes and stromal cells rather than functional necroptotic signaling.

Previous studies have demonstrated RIPK3 upregulation and MLKL phosphorylation in SJS/TEN skin lesions [25]. Our plasma-based findings extend this work and suggest that necroptosis-associated proteins may also be detectable systemically in the setting of severe epithelial injury. Elevated RIPK1 and RIPK3 levels may therefore reflect widespread tissue injury and inflammatory activation accompanying SJS/TEN.

These findings integrate with prior reports of increased IL-6, IL-15, IL-1β, IL-13, and granulysin expression in SJS/TEN plasma and blister fluid [10,11,12,13,19,20,28,34,35]. The coexistence of these inflammatory mediators with elevated RIPK1 and RIPK3 is consistent with overlapping inflammatory and cell-injury pathways, although mechanistic relationships cannot be established from the present study alone.

The concurrent elevation of RIPK1 and RIPK3 is biologically consistent with necroptosis-associated signaling pathways. RIPK1-mediated recruitment of RIPK3 promotes MLKL phosphorylation and downstream membrane disruption, DAMP release, mitochondrial ROS generation, and inflammasome activation [10,23,27,28,29,30,36,37,38]. However, these mechanisms were not directly evaluated in the present study.

Variability within the SJS/TEN cohort may reflect differences in disease severity, timing of plasma collection, inciting medications, and underlying immunogenetic susceptibility [4,5,6,7,8,9,10,11,12,13,14,15]. Prior studies have similarly demonstrated substantial interpatient variability in inflammatory cytokine expression among SJS/TEN patients [12,19,20]. Future studies incorporating clinical metadata such as the SCORTEN score, the culprit medication, and the timing of sampling will be important for clarifying these associations.

If validated in larger cohorts, circulating RIPK1 and RIPK3 may warrant further evaluation as exploratory biomarkers associated with systemic inflammatory burden in SJS/TEN. However, they cannot currently be considered mechanistic biomarkers or therapeutic targets.

This study has several important limitations, including a small sample size, heterogeneous pathological plasma controls, a lack of longitudinal sampling, and an absence of detailed clinical metadata. In addition, ELISA-based measurements quantify total circulating RIPK1 and RIPK3 but cannot distinguish phosphorylated active forms from inactive proteins or determine cellular origin. As a cross-sectional observational study, causal relationships cannot be established.

Nevertheless, these findings provide preliminary translational evidence of an association between SJS/TEN and circulating necroptosis-associated proteins and support further investigation in larger, longitudinal, and clinically annotated cohorts.

## 5. Conclusions

In this study, we observed elevated circulating levels of RIPK1 and RIPK3 in patients with SJS/TEN compared with controls. While these findings are consistent with prior tissue-based evidence implicating necroptosis-related pathways, they do not establish pathway activation or causality.

Several limitations should be considered, including a small sample size, heterogeneity of control groups, and limited clinical metadata. In addition, measurements of total protein levels do not capture phosphorylation-dependent activation states, which are critical for confirming necroptotic signaling.

Accordingly, our results should be interpreted as exploratory and hypothesis-generating. Future studies incorporating larger, well-characterized cohorts and functional or activation-specific assays will be necessary to determine the mechanistic and clinical significance of circulating RIPK1 and RIPK3 in SJS/TEN.

## Data Availability

The raw data supporting the conclusions of this article will be made available by the authors on request.
